# Big data analysis of human mitochondrial DNA substitution models: a regression approach

**DOI:** 10.1186/s12864-018-5123-x

**Published:** 2018-10-19

**Authors:** Keren Levinstein Hallak, Shay Tzur, Saharon Rosset

**Affiliations:** 1 Department of Statistics and Operations Research, School of Mathematical Sciences, Tel-Aviv University, 6997801 Tel-Aviv, Israel; 2 Braun School of Public Health and Community Medicine, The Hebrew University of Jerusalem, 9112102 Jerusalem, Israel

**Keywords:** Mitochondrial DNA, Substitution models, Regression, Partitioning, Context

## Abstract

**Background:**

We study Phylotree, a comprehensive representation of the phylogeny of global human mitochondrial DNA (mtDNA) variations, to better understand the mtDNA substitution mechanism and its most influential factors. We consider a substitution model, where a set of genetic features may predict the rate at which mtDNA substitutions occur. To find an appropriate model, an exhaustive analysis on the effect of multiple factors on the substitution rate is performed through Negative Binomial and Poisson regressions. We examine three different inclusion options for each categorical factor: omission, inclusion as an explanatory variable, and by-value partitioning. The examined factors include genes, codon position, a CpG indicator, directionality, nucleotide, amino acid, codon, and context (neighboring nucleotides), in addition to other site based factors. Partitioning a model by a factor’s value results in several sub-models (one for each value), where the likelihoods of the sub-models can be combined to form a score for the entire model. Eventually, the leading models are considered as viable candidates for explaining mtDNA substitution rates.

**Results:**

Initially, we introduce a novel clustering technique on genes, based on three similarity tests between pairs of genes, supporting previous results regarding gene functionalities in the mtDNA. These clusters are then used as a factor in our models.

We present leading models for the protein coding genes, rRNA and tRNA genes and the control region, showing it is disadvantageous to separate the models of transitions/transversions, or synonymous/non-synonymous substitutions. We identify a context effect that cannot be attributed solely to protein level constraints or CpG pairs.

For protein-coding genes, we show that the substitution model should be partitioned into sub-models according to the codon position and input codon; additionally we confirm that gene identity and cluster have no significant effect once the above factors are accounted for.

**Conclusions:**

We leverage the large, high-confidence Phylotree mtDNA phylogeny to develop a new statistical approach**.** We model the substitution rates using regressions, allowing consideration of many factors simultaneously. This admits the use of model selection tools helping to identify the set of factors best explaining the mutational dynamics when considered in tandem.

**Electronic supplementary material:**

The online version of this article (10.1186/s12864-018-5123-x) contains supplementary material, which is available to authorized users.

## Background

The human mitochondrial DNA (mtDNA) is a short circular haploid and non-recombinant chromosome, which is maternally inherited. It has different codons, replication, and proofreading mechanisms compared to the autosomal DNA [1–3]. The lack of recombination allows for a simpler analysis of the complex substitution process underlying the phylogenetic mechanism, compared to recombinant DNA. Specifically, all historical human mtDNA can be modeled using a common phylogenetic tree, with the leaves corresponding to extant individuals. Branchings in the tree describe separations of lineages that lead to different offspring today. The root of the tree can be designated as “mitochondrial Eve” [4]. The process of inferring the tree structure from sampled DNA sequences has been studied extensively in the literature, and methods such as maximum likelihood [5], maximum parsimony [6] and neighbor joining [7] were proposed and compared [8, 9]. Several software packages implementing these methods are publicly available [10, 11].

There are a few comprehensive mtDNA databases such as MITOMAP [12], mtDB [13] and Phylotree [14] that are regularly updated. In this work we use the highly trusted phylogenetic tree reconstructed by Phylotree. Phylotree has been updated several times throughout the years and currently (Build 17) consists of 24,275 sequences of extant individuals (reference files) and 5,437 internal nodes. Behar et al. [15] published a refinement of the tree which included a high confidence reconstruction of its root which they termed RSRS (Revised Sapiens Reference Sequence). In our work we use the most updated tree and the RSRS root, and assume it reliably describes the true phylogenetic history. Since the observed mtDNA sequences are located in the leaves, we can refer explicitly to the three sources of potential uncertainty in generating the list of substitutions inferred from the tree: First, we assume that the phylogeny of the tree specifying its topological structure as given by Phylotree is correct. This assumption was previously justified in several papers [15–18], where it was mainly supported by the sparsity of substitutions in branches in deeper layers of the tree and the resulting successful haplogroup estimations.

The second source of uncertainty relates to the ancestral sequences in the hidden layers of the tree. Most nodes can be confidently reconstructed by maximum parsimony, however, there may still be some uncertainty in the reconstruction [17].

Finally, considering the identity and number of specific substitutions along the branches, it is possible that several substitutions occurred at one site on the same branch, but the sequences in the nodes only contain the initial and final state of the site on this branch. For example, in a certain site there might be a C base transitioning to T, followed by a transversion to A. It may be the case that only the starting C and finishing A are estimated in the nodes’ sequences. This results in underestimation of the number of substitutions in that site by assuming only one *C* → *A* transversion occurred. A variety of similar events can happen, where multiple substitutions follow in a branch in the same site. Soares et al. [18] argue that in a long branch in the human mtDNA phylogeny, the probability that one of the highly mutated sites in mtDNA (site 152) has multiple substitutions on the same branch is below 1%. Since most of our analyses do not consider such fast sites, the probability of repeated unobservable substitutions is probably negligible. Finally, even were there multiple unobserved substitutions along one branch, these will generally result in slightly noisy observations to which the regression models we use are quite robust; subsequently, we feel confident in assuming all substitutions are specified by Phylotree. This greatly simplifies the statistical analysis as it requires no additional inference on latent information.

In this work we focus on genic regions in mtDNA, though our methods can be easily extended to other sub-regions. We take advantage of the large quantity of high quality mtDNA substitution data to perform comprehensive statistical modeling and find the best fitting model using the Akaike information criterion (AIC) [19]. Our data driven approach focuses on analyzing the significance of the effect of possible factors on the substitution rate. Relevant factors might include the current base/codon, the sub-region in which the site is located, its neighboring nucleotides and more. Assessing the importance of each factor can help in understanding the underlying biological principles affecting mtDNA substitutions. For example, if some genes (or sub-regions) are likely to follow the same substitution model, then from a biological point of view they are possibly functionally related and from a statistical standpoint we can aggregate these genes to gene-clusters to increase the power of future research.

### Our main contributions are as follows

1. We utilize the most updated Phylotree data and execute an exhaustive analysis on the effect of multiple factors on the substitution rates through both Poisson and negative-binomial regressions. We examine for each factor if it should be included as an explanatory variable in the substitution model, and/or if the substitution model should be partitioned according to it. The factors we examine include categorical factors that are not constant per site (for example: the input codon, amino acid, nucleotide, and context can vary at a given site along the tree). We incorporate these factors into the regression by adding for each factor an exposure term that represents the time spent in each possible value of the categorical factor, thus, allowing for non site-based partitions. We also examine if it is beneficial to model all substitutions together or separate them into transitions/transversions or synonymous/non-synonymous substitutions. To the best of our knowledge, our regression approach is a new statistical perspective on modeling DNA substitution data, allowing to quantify the significance of all factors simultaneously. A detailed description of our approach is given in the Methods section.
2. Our results show it is advantageous to model transitions and transversions together as well as synonymous and non-synonymous substitutions. We show that neighboring nucleotides should be included in the substitution model (as explanatory variables or partitioning factors) even when protein level constraints and the CpG pairs are taken into account. For protein coding genes, the substitution model should be partitioned into sub-models according to the codon position and input codon; each sub-model should include the direction of replication as an explanatory variable while genes identities should not be included in the model. A detailed description of these results is given in the Results section.
3. We apply a novel clustering technique on genes that is based on three similarity tests between each pair of genes as detailed in the Methods section. Our new method supports previously found gene functionalities.

### Previous works and points of interest

There is a large corpus of previous work on substitution models for DNA. Extensive literature considers a reversible continuous Markov chain model such as JC69 [20], F81 [5] and HKY85 [21]. These models describe the probability for every base to transition to every other base after a specific time duration using a rate matrix whose constraints differ between the possible models. When considering only the number of substitutions per site, models with substitution rates that are independent of the current nucleotide (such as JC69 and F81) induce a Poisson distribution on each site.

An important setting is when substitutions at each site have a Poisson distribution, but different sites have a different Poisson rate parameter. If the rate of each site is drawn from a Gamma distribution, the marginal distribution is Negative Binomial (NB), as was described and used by Tamura and Nei [22].

A different body of research deals with more specific testing of different factors and phenomena that could affect DNA substitution rates such as hot-spots [23], CpG pairs [24] and context (neighboring nucleotides) [25, 26]. For instance, context is considered an important factor in coding sequence non-randomness utilized for efficiency and accuracy in protein synthesis [27]. In their work, Aggarwala and Voight [25] showed that most of the variability in polymorphism levels in autosomal DNA can be attributed to the context, and used these results to detect irregularities correlated with neurodevelopmental and psychiatric disorders; indeed, our results also show that context is a significant explanatory variable, even when other explanatory variables are considered.

Johnston and Williams [28] study gene retention in mtDNA across eukaryotes and its relevant causes. In their work, they show GC content and protein hydrophobicity to be significant factors in mtDNA gene retention. These factors are perhaps most related to the evolutionary conservation score and protein domain factors [29] that we include in all sub-models as a regression effect.

Another relevant body of works by Zoller and Schneider [30, 31] investigates what are the most relevant substitution rate matrix parameters for codon and amino acid models by applying principal component analysis (PCA) on the empirically estimated parameters. Thus, they utilize a two-step approach of first estimating the rate for each site without using any explanatory factors, and then post-processing the estimates to expose the effects of such factors. Our approach, on the other hand, is based on modeling the rates while accounting for explanatory factors by considering many possible models and examining which ones fit best; we also concentrate on mtDNA.

Some papers consider the codon structure instead of relating directly to the bases. For example, Zaheri et al. [32] propose to model the codon substitution rate matrix using a Kronecker product of nucleotide substitution rate matrices. Several codon-substitution models based on the reversible continuous Markov chain model have been suggested, where usually the key parameter is the damping of non-synonymous transition rates [33]. In the regression models we test, we consider both the codon and the amino acid it encodes as relevant factors and test their effect on the substitution rate. Subsequently, we test whether the basic unit of the model should be a nucleotide, a codon or an amino acid, or several at once, while considering all options as viable. There is a vast literature comparing nucleotide, amino-acid and codon based models [34–37], and all but the last support codon based models. Indeed, our results agree that codons are the major basic unit required for inference on the number of substitutions.

Site-based partitioning of the substitution model into independent substitution sub-models implements the assumption that each group of sites has evolved under the same evolutionary process. Partitioning was applied ad hoc in several works, mainly according to codon position and genes [38–41]. Due to the intractable nature of performing an exhaustive search over all partitioning options, several partitioning search algorithms were developed, such as a hierarchical clustering method suggested by Li et al. [42], and the popular PartitionFinder open source program [43] which efficiently finds optimal partitions using a heuristic search algorithm. In this work we chose to implement an exhaustive search over all possible partitioning options for several reasons; first, heuristic searches are not guaranteed to find the optimal partitioning scheme [42], so an exhaustive search is always preferred, if computationally possible. Second, the previously mentioned methods perform site-based partitioning, allowing to partition over site based factors such as genes and codon position. However, factors that change along the tree in each site, such as nucleotide, amino acid, codon and neighboring nucleotides cannot be incorporated into this site-based partitioning method.

## Methods

### Data preprocessing

The substitutions’ data was obtained from the Phylotree website [14], where the tree was constructed so that only substitutions that were shared by at least three complete sequences were included (with a few exceptions). The substitutions A16182c, A16183c, C16519T/T16519C were not considered for phylogenetic reconstruction since they are mutational hot-spots, so their respective sites are excluded from the data. In the phylogeny tree there are 12,961 substitutions and 5,437 haplogroups. There are 1,113 haplogroups that have one representative mtDNA sequence (reference file) and 3,578 that have two (some haplogroups have none). We examined these sequences and found 20,696 substitutions that were not included in the tree (most of them singletons) and added them to the data, resulting in a total of 33,657 substitutions. The empirically observed transition/transversion ratio is 21.97 in the mtDNA overall and 22.4 in the genic regions. The empirically observed nonsynonymous/synonymous substitution ratio is 0.418.

The approach taken in this paper requires filtering the substitutions by additional parameters other than the site in which they occurred. For example, we are interested in filtering substitutions according to the neighboring nucleotides. These filtrations are achieved by first forming for every node in the tree the full mtDNA sequence it represents, and then storing the corresponding parameters for every site in each branch.

### Genes clustering

Some genes are known to have similar functionality [1], and genes that belong to the same “functional family” have similar names. For instance, there are two ATP genes in the human mtDNA (named ATP6 and ATP8), which encode subunits of ATP synthase. Aggregation of genes that follow the same substitution model is desirable, as it effectively reduces the number of distinct sub-models required to characterize the data, thus, reducing the degrees of freedom and improving the quality of the resulting estimates. Yet, blindly aggregating genes together by functionality can lead to aggregating genes with different substitution models, or to missing a previously unknown similarity in substitution models between genes of different functional families.

To better support any choice of aggregation, we have devised several similarity tests between genes. Each test examines the hypothesis that two genes share some mutational characteristics and returns an appropriate *p*-value. The combined results for all tests can either support or oppose the aggregation of every two genes. The tests are:

1. Compare the per-site substitution count of the two genes using the Kruskal-Wallis test [44].
2. Fit a NB model to the number of substitutions per site for each gene separately, and for the aggregation of the two genes. Compare the results using a Generalized Likelihood Ratio (GLR) test.
3. Perform a NB regression with the gene as an explanatory variable — its *p*-value corresponds to whether the genes should be joined.

Note that tests two and three are very much alike and test similar null hypotheses but they are not identical. If several genes behave identically with respect to the substitution model, we expect none of the hypotheses to be rejected between every pair of these genes. We applied each one of the three above mentioned tests to each pair of genes $$ \left(\left(\begin{array}{l}13\\ {}2\end{array}\right)=78\; pairs\right) $$ and compared the obtained *p*-values to a Bonferroni corrected critical value $\alpha =\frac{0.05}{78}$. A p-value lower than this threshold means that the null hypothesis that the two compared genes are “similar” (the meaning of this is different for each test) was rejected. We used the results to cluster together genes whose comparison yielded *p*-values higher than the critical value. The rule we applied for clustering genes was that in order to be joined, two genes must be “similar” under all tests. The resulting clustered genes’ groups are as follows (see the Results section for details):

1. ***NDCO*** *: ND1, ND2, ND3, ND4L, ND4, ND5, ND6, CO1, CO2, CO3*
2. ***ATP*** *: ATP6, ATP8*
3. ***CYB***

### Variables affecting the substitution rate

The models consider the following categorical factors:

1. Genes
2. Clustered genes as described in the previous section
3. The input nucleotide which was present before the substitution occurred (A/C/G/T)
4. The input amino-acid which was present before the substitution occurred (21 amino-acids)
5. The input codon which was present before the substitution occurred (64 codons)
6. The codon position (1/2/3)
7. The right and left neighboring nucleotides
8. Whether the site was part of a CpG pair and if so was it the first or second position
9. Directionality (indicator to whether or not the gene is located on the light strand; considering mtDNA protein coding genes its value is 1 only for ND6 and 0 for the other genes)

Additional site-based factors included in all models are evolutionary conservation as calculated by phyloP100way vertebrate (based on multiple alignments of 100 vertebrate species and measurements of amino-acid evolutionary conservation) [29] and protein family, transmembrane and low complexity protein domains as found by Bateman et al. [45]. These factors are numerical explanatory variables (not-categorical) previously evaluated per-site on the entire mtDNA.

### Poisson and negative binomial regressions

Poisson point process is a memoryless count process implying exponentially distributed waiting time between events. Assuming that the substitution process at a given site is a Poisson process requires the assumption that it is memoryless, i.e., that the number of substitutions in a time interval is independent of the number of substitutions in other, non-overlapping time intervals. The substitution rate, which we will denote here as*λ*, may however depend on both observed and unobserved variables. Examples of possibly relevant observed variables are gene, codon position and other variables mentioned in the previous subsection. When all relevant variables are observed, and the memoryless assumption holds, the substitution process can be modeled as a conditional Poisson process. Assuming that the dependence of log(*λ*)on the variables is linear, we model log(*λ*) using a linear combination of the input explanatory variables by applying a Poisson regression with a log link function. The optimal solution can be found through Fisher’s scoring method [46] or by convex optimization schemes (such as gradient descent).

It might be the case that in addition to the relevant observed variables, there are also latent variables (variables that were not deemed as relevant, or hidden variables that were not observed at all) affecting the substitution rate. In this case, the substitution process can be properly modeled as a conditional over-dispersed Poisson process.

If the over-dispersion is properly modeled by a Gamma distibution, the resulting substitution model follows a conditional NB distribution. This can be modeled using a NB regression, similarly to Poisson regression. The notion of underlying Gamma distributed rates for every site was previously considered by Tamura and Nei [22] and became a standard approach in inference over the phylogenetic tree [10, 11, 47].

We follow this view and perform both Poisson regressions and NB regressions to model the substitution rate at each site. If the Poisson model is less preferable (based on the AIC score of the models), then this means there are still missing relevant factors. In this case, a latent Gamma-like distribution accommodates for the uncertainty, resulting in a better fitting NB regression.

### Adding exposure to the Poisson and negative binomial regressions

We define a state *S* as a specific set of values of the explanatory variables discussed above. For example, the state *S* = {*Site* = 7765, *Codon* = *GAG*, *Neighbor* = *A*} corresponds to site 7765 with an input codon GAG, codon position 3 and right neighbor A. Note that once the site, codon, and neighbor are known, all other variables are also known (the site determines the relevant gene, clustered gene, codon position, directionality and the additional site-based factors; the codon determines the amino acid and when the codon position and neighbor are known the input nucleotide and CpG condition can also be determined). Subsequently, a substitution in the corresponding site of a state will change the nucleotide of that site, so the codon defining the state will change. For example, a transition from G to A in site 7765, which is in codon position 3 for the state given above will result in a new state.

Assuming the substitution process is a Poisson process in each site dictates that the expected number of substitutions is proportional to the amount of time spent in each state. Since a substitution changes the state, this kind of model is only viable when the rate is very small such that in each state it is highly unlikely that more than one substitution per branch can occur. Indeed, when looking on the observed data, less than 0.5% of the tree branches contain simultaneous substitutions in the same state at two different sites.

Therefore, we calculated the time spent in the tree in each state and added this time as an exposure variable; for instance, to include the input codon as an explanatory variable, we calculated for each site how much time was spent in each codon over the whole tree. While this could potentially augment the data times 64, in fact at each site there were no more than four different codons throughout the tree. Adding the time as an exposure variable to a regression with a log link function (such as the Poisson and NB regressions we applied) means that given the feature vector *x* (whose coordinates are composed of indicator variables for the categorical explanatory variables which were included in the regression), the regression finds the optimal *β* coefficients such that log(*λ*) = *β*^*T*^*x* + log(exposure time), forcing *λ*, the expected number of substitutions to be proportional to the time spent at the state as required.

When modeling separately transitions/transversions, we generate separate regressions: one with the number of transitions as a response, and the second with the number of transversions; both regressions have the same input feature vector *x* but differ in the resulting coefficients vector*β*. The same is true for synonymous and non-synonymous substitutions.

In addition, we add an exposure variable to the case where we separate synonymous and non-synonymous substitutions. When the number of synonymous substitutions is modeled separately, there are states in which a synonymous substitution is impossible. Consider for example the codon GTT that codes for the Valine (Val) amino acid: in its first and second position there are no possible synonymous substitutions, while in its third position there are three possible synonymous substitutions — one transition (GTT → GTC) and two transversions (GTT → GTA, GTG). In the same manner, we can calculate the number of possible non-synonymous substitutions for each codon position and divide them into possible transitions and transversions. We expect the number of synonymous substitutions at each state to be proportional to the number of **possible** synonymous substitutions with respect to the number of possible transitions and transversions. Subsequently, when the number of synonymous/non-synonymous substitutions was modeled separately, we added the number of possible synonymous/non-synonymous substitutions as an exposure explanatory variable, when taking into account the number of possible transitions/transversions and weighting them according to the transversions/transitions ratio empirically found in the data(*r* = 0.04551103). In these cases the regressions for synonymous substitutions find the optimal*β*coefficients such that: log(*λ*) = *β*^*T*^*x* + log(exposure time) + log(#*A* + *r* ⋅  # *B*)*,* where #*A* is the number of possible synonymous transitions and #*B* is the number of possible synonymous transversions. Note that our exposure correction does not increase the rate at which substitutions occur, but simply adjusts the weighting between the rates of synonymous and non-synonymous substitutions. This method also accounts for the special case where no substitutions are synonymous (or no substitutions are non-synonymous) by setting the exposure to zero.

### Time estimation

To find the exposure of each factor’s value, we require an estimate of the time-length of each branch in the tree (or alternatively, the time of each branching event in the tree). As proposed by [48], we used a Poisson regression with an identity link function for time inference on all nodes in the tree. Substitutions that occurred in the tips of the tree were assigned the time *t* = 0. Notice that the assigned times are uncalibrated — since the exact timing of any branching in the tree is unknown, only the proportions between timings can be inferred. This also implies that all obtained substitution rate estimates are relative — all of them can be multiplied by a constant and all time estimates divided by the same constant without changing the likelihood. Notice that ideally we should have incorporated the estimation error term into the regression to account for additional variance, this was not done due to the difficulty of obtaining reliable confidence intervals in the said approach. Additional file 1: Table S1 contains uncalibrated time estimations for each node in the Phylotree dataset.

### Exhaustive search algorithm

For protein coding genes we examined 31,185 models composed of three different inclusion options for each categorical variable. The number of examined models is not three to the power of the number of factors since there are many exceptions where models are removed when they are contained in another model. For instance, we did not consider models with both codon and amino acid factors, nor models with both codon and input nucleotide factors, since the codon variable contains the information given by both; hence, the number of possible models resulting from the variation of the codon, amino acid and input nucleotide inclusion options is 11 instead of 27. For the same reason, we did not consider models with both the gene and clustered gene, so the number of possible inclusion options for the gene and clustered genes variables is five instead of nine. When the data was partitioned according to the codon position, the right and left neighbor variables were coerced to follow the same choice of inclusion. Hence, the number of possible inclusion options for the codon position and right and left neighboring nucleotides is 21 instead of 27. We also note that for models with the codon as an explanatory variable which were partitioned according to the codon position we included only the right and left neighboring nucleotides outside of the codon (left neighbor for the first codon position, right neighbor for the third codon position and none for the second codon position). Finally, some of the resulting models were removed from the analysis since the inclusion of certain factors contained all information on the value of another factor (for example, genes contain information about the directionality since only ND6 has opposite directionality). Such models were removed from Table 2.

Overall we examined 11 ⋅ 5 ⋅ 21 ⋅ 3^3^ = 31, 185 models, calculated as the product of the number of options for each variable as specified before: codon / amino acid / input nucleotide, gene / clustered gene, codon position / neighboring nucleotides, directionality, CpG pair status, and the possible response models.

For the rRNA genes and control region, we do not include the direction of replication since all sites have the same directionality. Subsequently for these we inspect 486 models each - three options for CG pair, right and left neighbors, sub-regions/genes and input nucleotide and two options for the response (transition\transversion or all). For the tRNA genes, there are three times as many models (since the direction of replication is included) amounting to 1,458 models.

We note that this exhaustive search over all options was necessary since the inclusion of each categorical variable may affect the other variables. For example, if the model is partitioned into sub-models according to the input codon, it may no longer be statistically useful to divide into sub-models according to the gene.

We finish this section with some technical remarks: The models we examined are composed of 8,671,658 sub-models when considering the partitions according to all categorical variables; so we performed 8,671,658 NB and Poisson regressions, out of which 51,333 (0.006%) NB regressions and 18,074 (0.002%) Poisson regressions did not converge. Since all sub-models are needed for calculating the AIC score of each model, it was necessary to include the models that did not converge. To do so, we assigned these models likelihoods according to a Poisson distribution with a parameter *λ* equal to the number of observed substitutions in each state and the degrees of freedom were taken to be the number of different states (rows) in the sub-model. In addition, there are 3,212,855 sub-models that are composed of one state or include zero substitutions, whose likelihood and degrees of freedom were taken to be one (except for sub-models that included one state with at least one substitution whose likelihood was calculated according to a Poisson distribution). The total running time amounts to less than three days on a 64 core cluster.

## Results

### Genes clustering

In the Methods section we suggest several similarity tests to examine the null hypothesis that two genes are similar. These tests were applied to each pair of genes and their *p*-value was compared to a Bonferroni corrected critical value of$\alpha =\frac{0.05}{78}$. Our results are summarized in Table 1. Under all tests, the null hypothesis could not be rejected for pairwise comparison of genes from the group *ND1–6* and *CO1–3,* suggesting that they should be clustered together. From here on, we shall refer to this group of genes (*ND1–6* and *CO1–3*) as *NDCO*. *ATP6* and *ATP8* can also be joined under all tests and will be referred to as *ATP*. The combined groups: *NDCO, ATP* and *CYB* are used as a “clustered genes” explanatory variable in the modeling process. We note that our clustering procedure was made without taking into consideration the biological functionality underlying the different genes [1]; subsequently, the tests we performed further support the previously found biological partitions.

**Table 1:**
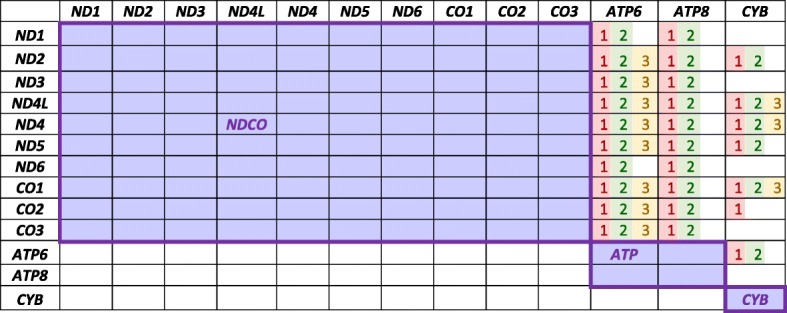
The results of the clustering tests on the different pairs of genes; each cell in the table contains the indices of the null hypotheses which were rejected (ranging from 1 to 3). Empty cell means that none of the null hypotheses were rejected, and hence the genes are similar. Due to symmetry, cells below the diagonal are not marked. Tests 1–3 compare the substitution count distribution through (1) Kruskal-Wallis, (2) negative-binomial model and (3) negative-binomial regression. The resulting clusters by our tests are (NDCO: *ND1–6, CO1–3*), (ATP: *ATP6, ATP8*) and *CYB*

### Exhaustive search algorithm

We aim to find the best substitution model, where we use AIC as a criterion for comparing models. We use the term model to denote a specific choice of inclusion for each one of the categorical variables that might affect the substitution rate as listed in the Methods section. The inclusion options for each variable are as follows: for each categorical explanatory variable we can choose between (1) not including it in the model, (2) including it as an explanatory variable or (3) partitioning the data according to it and building a separate sub-model for each part of the data. Each model is therefore composed of a varying number of sub-models according to its specific inclusion options. For example, a model that partitions the data according to the codon position and does not include all other variables will be composed of three sub-models — one for each codon position. We calculate the AIC score of the full model as the sum of AIC scores of all of its sub-models (this is possible since the AIC score is linear in the log-likelihood and in the number of parameters).

We calculated the AIC for each one of these models with the response as (1) the sum of all substitutions, (2) two separate models, one for the number of synonymous substitutions and the other for the number of non-synonymous substitutions and (3) two separate models, one for the number of transitions and the other for the number of transversions.

### Protein Coding Genes

Overall, we examined 31,185 models and ordered them by their minimal AIC score (obtained by either Poisson regression, or NB regression); the top 20 results of our algorithm are given in Table 2 and all results appear in Additional file 2: Table S2. For each model we specify which factors partitioned the model into sub-models (marked as ), and which factors were included / not included in all the resulting sub-models (marked as /  correspondingly).

**Table 2:**
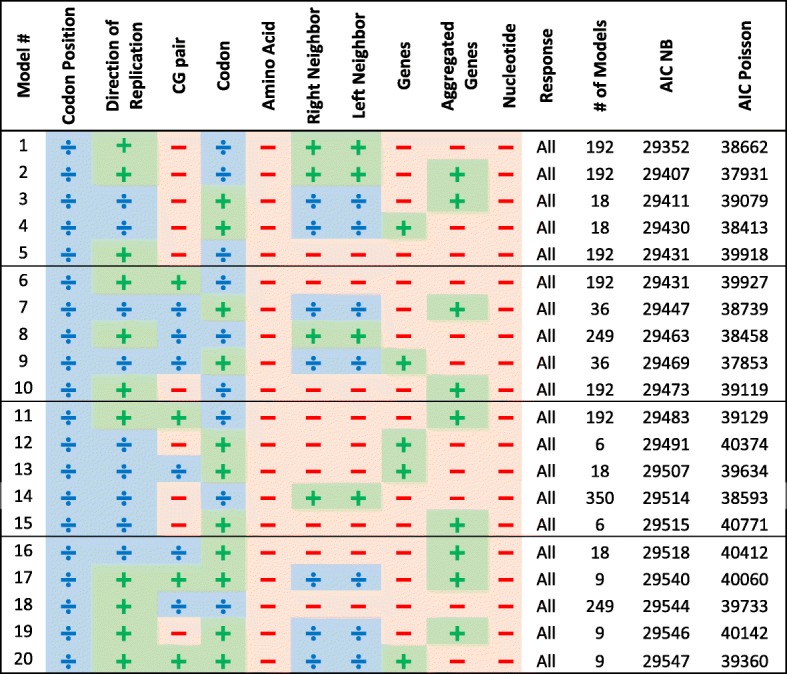
Top 20 models for protein-coding genes ordered by their minimal AIC score (out of Poisson and NB AIC scores). Each categorical variable obtains one of the following signs: ,  and  that mark partitioning, inclusion as an explanatory variable and omitting the variable correspondingly. The value “All” in the response column means all substitutions were modeled together in these top models (and not separately for transitions/transversions and synonymous/non-synonymous substitutions

All top 20 models:

- Partition into sub-models according to the codon position
- Include the input codon (as an explanatory variable or partition according to it) and subsequently do not include the input amino acid and input nucleotide
- Include the direction of replication (as an explanatory variable or by partition)
- Do not divide the substitution’s type to separate models for transitions/transversions or synonymous/nonsynonymous substitutions.
- Result in a substantially lower AIC score for the NB model compared to the Poisson model

Also, most of the top 20 models include the right and left neighbors.

The model with the lowest AIC score is composed of 192 sub-models with different codon positions and input codons (64 sub-models for each of the 3 codon positions, hence 192 = 64·3 sub-models). The explanatory variables in each model are the right and left nucleotide neighbors, the directionality and the additional site-based variables that were included in all models.

Another interesting result relates to the subject of model partitioning, which was discussed in the introduction. As previously mentioned, there are several algorithms that heuristically find best fit partition schemes [42, 43]. While these algorithms necessarily result in site-based partitions, our results show that the model with the lowest AIC score partitions the data according to the input codon in addition to the codon position. Indeed, previous works [38, 40] have shown that partitioning the substitution model according to the codon position is beneficial. However, partitioning according to the input codon with additional conditions was not considered yet due to practical limitations [49], though it is shown here to be advantageous.

We examined the effect of neighboring nucleotides and CpG pairs as explanatory variables and found that neighboring nucleotides have a significant effect and should be included as explanatory variables in the model. Our definition of neighboring nucleotides refers to nucleotides outside of the codon when the input codon is included in the model, so for the first codon position we take into account only the left neighbor, for the second codon position no neighbors are considered and for the third codon position only the right neighbor is considered.

Comparing models 1 and 5 in Table 2 (that differ only by the inclusion of the neighboring nucleotides) using a GLR test allows to examine the *H*_0_ hypothesis that the neighboring nucleotides have an insignificant effect on the substitution rate. The result (*p*-value *<*1*e*^− 12^) shows that the neighboring nucleotides have a significant effect on the substitution rate. However comparing models 5 and 6 (that differ only by including the CpG trait as an explanatory variable) using a GLR test shows that the CpG trait effect alone is insignificant. Note that the leading model partitions the data according to the input codon and codon position, and also includes neighbors, so it technically contains the CpG trait information. To conclude, our results show that neighboring nucleotides have a significant effect on the substitution rate and should be added as an explanatory variable, whereas including the CpG trait alone is not enough.

### Control region, rRNA and tRNA genes

Similarly to the protein-coding genes, we applied our exhaustive search algorithm on the rRNA and tRNA genes, and on the control region separately. Our top 10 results are given in Tables 3, 4, 5 and all results appear in Additional file 3: Tables S3, Additional file 4: Tables S4, Additional file 5: Tables S5 correspondingly. Note that for these regions, many of the previously specified explanatory variables are no longer relevant as these regions are not composed of codons. In addition, the response is no longer divided to synonymous/non-synonymous substitutions.

**Table 3:**
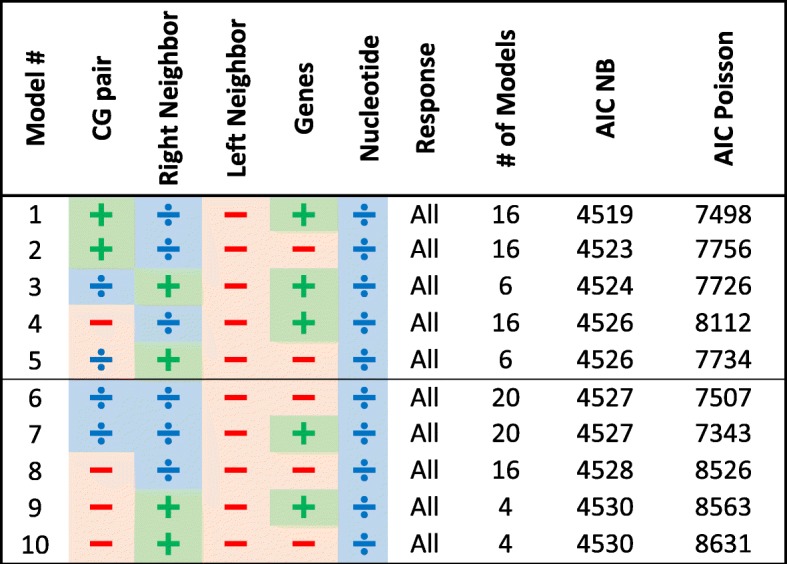
Top 10 rRNA models ordered by their minimal AIC score (out of Poisson and NB AIC scores)

**Table 4:**
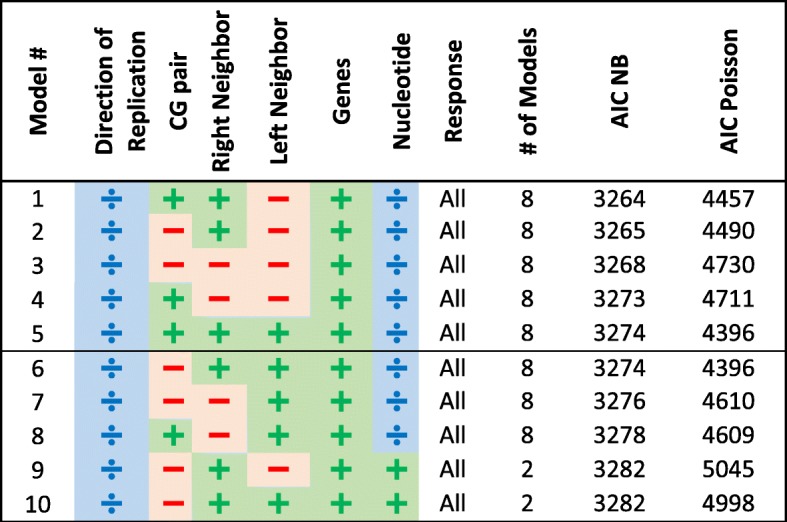
Top 10 tRNA models ordered by their minimal AIC score (out of Poisson and NB AIC scores)

**Table 5:**
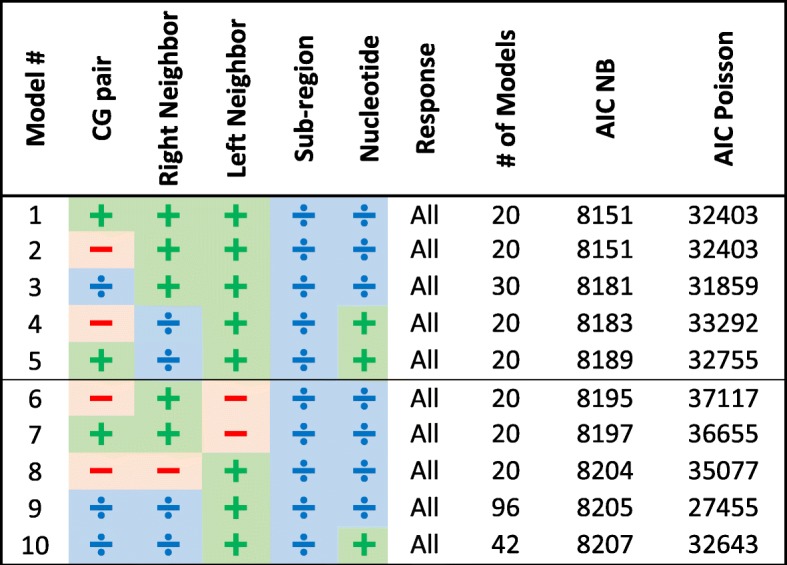
Top 10 control region models ordered by their minimal AIC score (out of Poisson and NB AIC scores)

In all Tables 3, 4, 5 the separation between transitions and transversions leads to inferior models compared to models that analyze the combined set, similarly to the results on the protein-coding genes. The leading 50 models in all tables include the input nucleotide either as a partitioning factor (mostly) or as an explanatory variable.

Looking at the leading 10 models for the rRNA we can see that only the right neighbor affects the model while the left neighbor is left out. For the rRNA, there are only two genes (12S and 16S) and these do not have a conclusive effect. As for the tRNA, we can see that the direction of replication is a partitioning factor, and that the genes constitute an explanatory variable. For the control region, instead of genes we observe that sub-regions (Hyper-Variable Segments (HVS) 1–3 and the sub-regions between these) constitute an important partitioning factor.

### Poisson Vs. negative binomial regression

The leading models show AIC scores that are substantially lower for NB regression than for Poisson regression. As stated before, NB regression is preferable when there are missing relevant factors, and a latent Gamma-like distribution replaces the uncertainty, resulting in a more appropriate NB regression; thus this indicates that there might be additional factors we have not considered that affect the substitution rate. Such factors can be for example the clade in which the substitution occurred, the actual time if the molecular clock assumption is not valid or perhaps an inherent randomness suggesting that a conditional NB model (or perhaps another model that takes this randomness in substitution rate into account) is indeed preferable compared to a conditional Poisson model.

It is important to note that even if the leading model had a lower AIC score for the Poisson model than the NB model, it would still not mean necessarily that we have found all relevant factors. To see why, consider for example model #258 as ranked by the AIC score in the protein coding genes analysis (which appears in Additional file 2: Table S2): this model has the lowest Poisson AIC score out of all models and its NB AIC score is significantly higher than its Poisson AIC score (*AIC* = 30*,*049 for the Poisson model and *AIC* = 48*,*021 for the NB model). The model partitions the data according to codon positions, directionality, and genes and includes the CpG pair trait and input codon as explanatory variables. If we had not considered the first 257 models, this would have been our leading model, suggesting that we have considered all relevant factors. However, this is clearly not the case as our leading model has a lower AIC score, and its AIC score is lower for the NB regression.

## Discussion

Despite the extensive research in the field, there is limited understanding of the factors affecting substitution rates in various DNA modalities, and specifically in mtDNA. In our view, the reason for that is relatively small amounts of data, compared with the possibly large number of degrees of freedom stemming from the various possible factors affecting the substitution rate: site location, haplogroup association, non-stationarity, codon properties, context, and latent biochemical information. In this paper, we utilized the large, reliable mtDNA phylogeny in Phylotree to tackle this problem using proper statistical tools.

Unlike continuous time Markov chain models which specify the transition rate between every two instantiations of the basic model units (nucleotides, codons or amino-acids), we model directly the distribution of the substitutions count in a time interval, and its dependence on possible sets of observed factors. This formulation allows for simple Poisson/NB regressions to simultaneously consider combinations of variables participating as either partitions or as explanatory variables. We can thus choose the most suitable model by comparing the AIC scores of all models considered.

For the protein-coding genes, the model with the lowest AIC score includes partitions according to the input codon and the position inside the codon. Neither the amino acid, nor the input nucleotide, were enough to hold all information required to model the substitution process. In particular, the neighboring sites have a significant effect in setting the substitution process rate coefficients. We note that the observed context’s significance refers to neighboring sites adjacent to the codon (and not inside the codon). This significance remains even when the CpG trait is considered as an explanatory variable, so the CpG trait alone is insufficient to explain the context effect. Subsequently, in future work an expanded context should be considered as well. The origin of the detected effect described herein of neighboring sites cannot be explained by protein level constraints and we speculate it is related in a different manner to the nature of mutagenesis in mtDNA, perhaps through the replication mechanism. For example, an imperfect replication process by mtDNA polymerase γ (POLG) that relates to the neighboring sites was suggested to be responsible for the majority of mtDNA point mutations [50]. Nonetheless, the specific biological or chemical mechanism causing this effect is yet to be identified.

As far as we are aware, the direction of replication, was never considered explicitly as an explanatory variable in previous work. This could be since genes are commonly considered as explanatory variables and thus implicitly include the direction of replication. Our results show its effect can be captured sufficiently in an additive fashion, simplifying the model.

Interestingly, the genes themselves were not significant enough, which somewhat discourages the notion of purifying selection. Were the substitution rate affected by the functionality of the gene itself, then probably the more crucial genes would have shown a reduced substitution rate.

For non protein-coding genes (i.e., the control region and the tRNA and rRNA genes), we have also obtained some interesting results. The input nucleotide consistently appears in leading models as a partitioning factor, which means every nucleotide is entitled to a different sub-model; this is also true for the direction of replication in the tRNA genes. Another intriguing result can be seen in the leading rRNA models, where the right neighbor is a significant factor in determining the substitution rate, but the left neighbor is not included in any of the leading models.

The statistical approach we present here can be further applied to autosomal DNA, with appropriate adjustments considering its different properties; the recombination process in autosomal DNA challenges the possibility of finding a clear phylogeny allowing to track substitutions over time. However, its substitution rate is much slower compared to the mtDNA and is almost unique-event polymorphism (UEP). Approximating the substitution rate to be UEP, it should be possible to use logistic regression to model the probability that a substitution occurred over time, allowing to examine which factors are significant in the autosomal substitution process.

## Conclusions

A good understanding of the factors affecting the genomic substitution rate is critically important to many genomic and medical applications. These factors can include biological effects like selection, biochemical effects related to sequence and others. In this paper, we propose a statistical approach for multi-factor analysis of the substitution rate in phylogenetic trees. Our method provides a ranking of numerous discriminative models which can be used to simultaneously infer the importance of different subsets of factors. We applied our method to the Phylotree data-set of human mtDNA and investigated the effect of different factors on substitution rates within mtDNA. Our major conclusions include the critical role of both codon identity and codon position, as well as an independent effect of neighboring nucleotides on substitution rate. After these effects are taken into account, other factors like the identity and role of different genes do not have a significant effect on rates. We note that previous studies have suggested that gene identity affects substitution rate, probably due to not accounting for factors like codon composition.

Beyond the specific value of our conclusions, our study is unique in considering a huge space of possible models and combinations of affecting factors, and selecting between all of them in a principled, data-based manner using formal statistical inference. This gives renewed confidence in our conclusions, and supports future use of our methodology on other types of genetic data.

## Additional files

Additional file 1: **Table S1.** Uncalibrated time estimations for each node in the Phylotree dataset. (XLSX 177 kb)

Additional file 2: **Table S2.** All protein coding genes models ordered by their minimal AIC score. (XLSB 1472 kb)

Additional file 3: **Table S3.** All rRNA models ordered by their minimal AIC score. (XLSX 65 kb)

Additional file 4: **Table S4.** All tRNA models ordered by their minimal AIC score. (XLSX 178 kb)

Additional file 5: **Table S5.** All control region models ordered by their minimal AIC score. (XLSX 64 kb)

## Abbreviations

*AIC*: Akaike information criterion
*ATP*: ATP6, ATP8
*ATP6, ATP8*: ATP synthase subunit 6 and 8 genes
*CO1, CO2, CO3*: Cytochrome c oxidase subunit 1, 2, and 3 protein genes
*CYB*: Cytochrome b gene
*GLR*: Generalized Likelihood Ratio
*HVS*: Hyper-Variable Segment
*MtDNA*: Mitochondrial DNA
*NB*: Negative Binomial
*ND1, ND2, ND3, ND4L, ND4, ND5, ND6*: NADH dehydrogenase subunit 1–6, 4 L genes
*NDCO*: ND1, ND2, ND3, ND4L, ND4, ND5, ND6, CO1, CO2, CO3
*PCA*: Principal component analysis
*POLG*: Polymerase γ
*RSRS*: Revised Sapiens Reference Sequence
*UEP*: Unique-event polymorphism
*Val*: Valine amino acid
